# ImmTACs: bi-specific TCR-anti-CD3 fusions for targeted tumour killing

**DOI:** 10.1186/2051-1426-3-S2-P299

**Published:** 2015-11-04

**Authors:** Zoe Donnellan, Giovanna Bossi, Jane Harper, Joseph Dukes, Nathaniel Liddy, Samantha Paston, Tara Mahon, Peter Molloy, Malkit Sami, Emma Baston, Brian Cameron, Andrew Johnson, Annelise Vuidepot, Namir Hassan, Bent Jakobsen

**Affiliations:** 1 Immunocore Ltd., Abingdon, UK

## 

Tumour-specific T cells have the potential to eradicate cancer. However anti-tumour immunity is limited by low TCR affinities, MHC downregulation by cancer cells and an immunosuppressive tumour microenvironment. As most tumour-associated antigens are auto-antigens, tumour specific T cells with high affinity TCRs are likely to be deleted in the thymus. To overcome these limitations we have developed novel immune mobilising monoclonal TCRs against cancer (ImmTACs). These are soluble bi-functional molecules comprising of a high affinity TCR fused to an anti-CD3 scFV domain. ImmTACs recognise specific tumour antigens presented by MHC class I and re-direct T cells to mediate tumour killing.

Our first clinical candidate, IMCgp100, is specific for the melanoma-associated antigen gp100. The engineered TCR portion of the drug targets and binds the gp100280-288 epitope, which is overexpressed and presented by HLA-A2 on the surface of melanoma cells. The anti-CD3 scFv portion captures and re-directs T cells to kill the melanoma cells, while normal antigen-negative cells are not affected. IMCgp100 is currently in Phase I/IIa clinical studies in patients with advanced malignant melanoma. The maximum tolerated dose has been established and the drug is well tolerated with evidence of tumour reduction. Analyses of patient peripheral blood and serum samples show T cell mobilisation and transient drug-mediated increases in various cytokines and chemokines, some of which are reported to play a key role in anti-melanoma responses.

Here we present *in vitro* data which support the observations made in the clinic. IMCgp100 re-directs T cells from late stage cancer patients to target melanoma cells exhibiting HLA down-regulation, even in the presence of high numbers of regulatory T cells. Target cell killing is observed within hours and is specific for gp100. In addition, killing is associated with the release of various pro-inflammatory cytokines and chemokines, as well as cross-presentation of gp100 and other melanoma-associated antigens by dendritic cells. Thus, IMCgp100 demonstrates the potential to elicit potent short term responses and trigger longer-term anti-melanoma activity *in vivo*. These data support the potential of IMCgp100 as an effective and durable treatment for malignant melanoma. Overall these data present ImmTACs as a potent targeted immunotherapy.

